# Combined endocrine and exocrine tumours of the pancreas

**DOI:** 10.1186/1477-7819-5-103

**Published:** 2007-09-14

**Authors:** Ahmed Alzaraa, Valeri Udom, Husam Mousa, Abdulhalem Alzein, Abduljalil Benhamida, Neha Dalal

**Affiliations:** 1 Department of General Surgery, Tameside General Hospital, Manchester, UK; 2 Department of Radiology, Tameside General Hospital, Manchester, UK; 3 Department of Histopathology, Tameside General Hospital, Manchester, UK

## Abstract

**Background:**

Cystic neoplasms of the pancreas comprise 10%–15% of pancreatic cystic lesions, with the serous cystadenoms being the commonest. The association of exocrine and endocrine tumours of the pancreas unrelated to Von Hipple Lindau disease is very rare. Very few cases have been reported in the literature. We present another case of both these tumours in one patient.

**Case presentation:**

A female patient was seen in the surgical clinic for a pain in the right groin. Clinical examination and investigations confirmed a diagnosis of combined endocrine and exocrine tumours of the pancreas. She underwent surgery and is under regular follow-up in the surgical clinic.

**Conclusion:**

Biphasic differentiation of pancreatic stem cell during embryological development could happen and may result in combined endocrine and exocrine tumours of the pancreas. Imaging studies are excellent in diagnosing theses lesions. Surgery has a central role and could be curative.

## Background

The coexistence of endocrine and exocrine tumours of the pancreas is very rare. To our knowledge, only seven similar cases have been reported in the literature [1-6]. Here, we present the 8^th ^case with the radiological and histological findings.

## Case presentation

A 73 years-old lady was seen in the clinic in August 2006 for a right inguinal pain which had been present for a few months. She had no significant past medical history. Abdominal examination revealed a mass in the left upper quadrant. There were no other physical findings.

**Figure 1:**
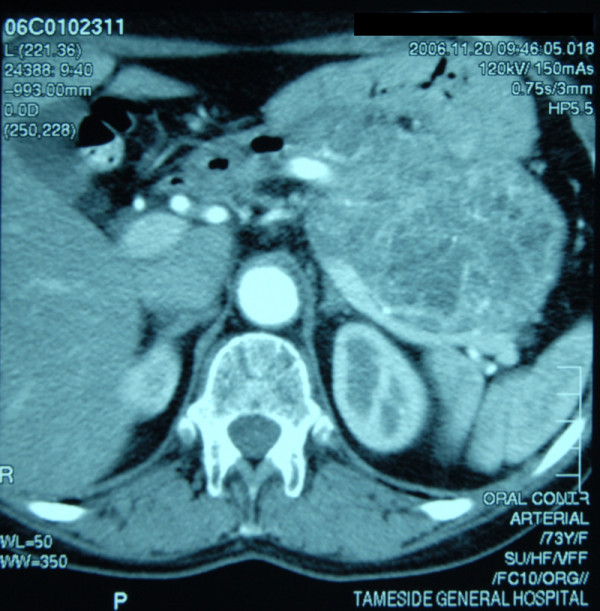
CT abdomen showing the mass arising from the body and the tail of the pancreas and extending into the splenic hilum.

Computed tomography (CT) of the abdomen confirmed a 12.3 cm × 8.2 cm mass arising from the body and the tail of the pancreas and extending into the splenic hilum (Figure 1). There was no lymphadenopathy involving the porta hepatis or the retroperitoneum. There were large simple cysts in the liver and the kidney. There were no other features of disseminated malignancy seen. Gastric endoscopy revealed an external mass pressing on the greater curvature of the stomach. CT chest was normal. The patient underwent tumour excision together with distal pancreatectomy and splenectomy in December 2006.

**Figure 2:**
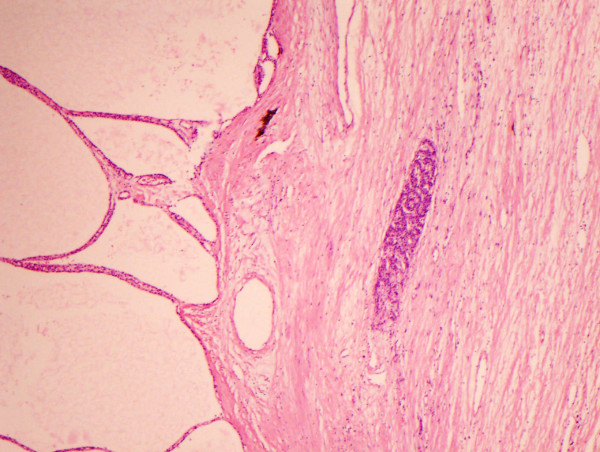
A low power view of the cystic spaces lined by cuboidal serous epithelium representing exocrine tumor with a cribriform sheet of endocrine tumor on the Right (×10).

**Figure 3:**
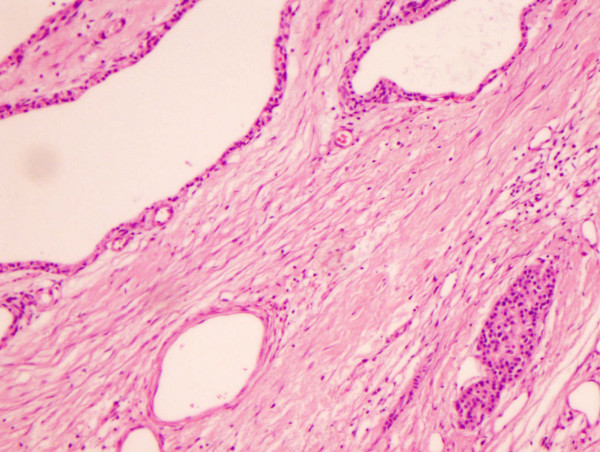
Cystic spaces on the left lined by serous type epithelium with a small sheet of endocrine tumor composed of small uniform cells with round nuclei (×20).

Histopathology report macroscopically showed a cystic mass measuring 13 × 10 × 10 cm and weighed 518 gm. The cut surface was spongy and multinodular. Microscopically, the tumour was composed of multiple cystic spaces lined by low columnar-cuboidal serous type epithelium with fibrocollagenous walls (figure 2). There was a focal papillary pattern. No significant cytological atypia or mitosis was seen. Within the stroma, there were nests of cords and trabeculae of small monotonous cells with speckled chromatin (figure 3). Immunohistochemically, these cells were positive for neuroendocrine markers; synaptophysin, chromogranin and neuron specific enolase. The appearance, therefore, represent a typical microcystic serous cystadenoma with an associated neuroendocrine tumour, probably of pancreatic origin and of uncertain malignant potential. The spleen was removed at the same time and there was extensive infiltration of the fibrocollagenous tissue at the splenic hilum by the neuroendocrine tumour.

The patient has not shown any sings of recurrence and is under regular follow-up in the surgical clinic.

## Discussion

Cystic neplasms comprise 10%–15% of pancreatic exocrine lesions [2]. They include a spectrum of benign, malignant and borderline neoplasms, of which, serous cystadenomas are most frequent. Pancreatic Endocrine Tumours (PETs) are rare (1 per 100,000 population), thought to originate from immature stem cells that are part of the neuroendocrine system. They are classified into functioning (70% of cases) if responsible for a clinical syndrome related to hormonal release, or non-functioning (15–30% of cases) if there are no hormone related symptoms [7] and may cause obstructive symptoms of the biliary tract, duodenum, gastrointestinal bleeding or abdominal mass. The commonest PETs are insulinomas and gastrinomas. The correlation between the morphological features and the clinical behaviour of PET is poor, and only the existence of metastatic disease indicates malignancy [5,6]. Virtually always PETs are treated surgically as they have significant malignant potential [5], and if the symptoms are not controlled, chemotherapy may be tried but response is limited. The coexistence of pancreatic exocrine and endocrine tumours is very rare. The first case was described by Keel et al in 1996 [6]. Embryologically, endocrine and exocrine pancreas develop from the endodermal lining of the duodenum, therefore, the potential of pancreatic ductal stem cells for biphasic differentiation along either glandular epithelial or neuroendocrine pathways is not unexpected [5,6]. Indeed, biphasic differentiation is observed in many different pancreatic disorders, both benign and malignant, and can happen in two patterns. The first pattern of mixed differentiation produces tumours showing a combination of PET-like features and features of either ductal or acinar cell. The second pattern is manifested by the concurrent development of distinct epithelial and neuroendocrine cells within the same tumour [5].

Cystic neoplasms are a heterogeneous group of tumours with a real problem regarding differential diagnosis between neoplastic and inflammatory lesions owing to the similarities in the clinical presentations and in the characteristics visualized in imaging studies [1,3,4]. The WHO classification of exocrine tumours of the pancreas is based on the histopathological features of the epithelial wall, which are the main factor in the differential diagnosis with cystic lesions of the pancreas [4].

Women in their 50s seem to be the population most affected by both endocrine and exocrine tumours and any portion of the pancreas can be involved, but they are most frequently detected in the pancreatic head [4,7]. The association of endocrine pancreatic tumour with cystadenomas is already known in patients with Von Hipple Lindau (VHL) disease, but the same association unrelated to VHL is quite rare, but not exceptional. In VHL, the pancreas is involved in up to 77% of patients, 12.3% serous cystadenomas, 12.3% by endocrine tumours and in 11.5% by combined lesions [1].

Clinically, cystic neoplasms are slow growing with no relevant symptoms or signs. When clinical sings are present, they may help pinpoint the organ of origin but never the type of pathology. Abdominal discomfort is the most common symptom. Weight loss, palpable mass, jaundice and obstruction of the upper gastrointestinal tract are rare and may be correlated to an extensive growth of the lesion [4]. More than 1/3 of these lesions are incidentally detected by abdominal ultrasound or CT scan performed for the evaluation of other condition [1,3,4]. Very little information is available on the growth rate of serous cystadenomas and on the likelihood of these lesions [3].

CT and Magnetic Resonance Imaging(MRI) are excellent tests for cystic lesions of the pancreas, not only for the initial detection of a lesion but also for the characterization of such lesions by visualization of the calcification of the cyst wall, septa, mural nodules, possible showing of communication between a cyst and the pancreatic duct, and findings suggestive of pancreatitis. The presence of a central scar visualized with the use of CT or MRI is a highly diagnostic feature that is found in about 20% of serous cystadenomas [3].

Management of these lesions has not been standardised, is evolving and depends on many factors such as the presence or absence of symptoms, surgical risk of the patient, age, type of the tumour, its location and size. The proper evaluation and subsequent management of this disease in patients without symptoms have not been fully defined [1,3]. The role of surgery is central because it could be curative when complete resection is possible [4]. PET arising in association with a serous cystadenoma may reinforce the recommendation for surgical therapy of cystic tumours of the pancreas as mixed tumours may be present [5]. The current death rate among patients undergoing pancreatic resection in specialized centres is less than 2% [3].

## Conclusion

Combined endocrine and exocrine tumours of the pancreas are very rare. The potential of pancreatic ductal stem cells for biphasic differentiation along the embryologic developmental line of exocrine and endocrine pancreas could happen. Cystic lesions of the pancreas are asymptomatic and mainly discovered incidentally. Imaging studies such as CT and MRI are excellent tests which help diagnose these lesions. The role of surgery is central in managing these tumours, especially if both endocrine and exocrine tumours coexist, and could be curative if complete resection is performed.

## Competing interests

The author(s) declare that they have no competing interests.

## Authors' contributions

AA Performed literature review, drafted and revised manuscript. VU Evaluated radiological features and contributed to radiological part of manuscript, AE Carried out initial assessment of the patient and helped in draft of manuscript. HM Contributed to the concept of the report AB Operated on the patient and corrected the manuscript for it scientific content. ND Evaluated histopathological features and contributed histological part.

All authors read and approved the final manuscript.
